# Genome-wide gene expression analysis in the placenta from fetus with trisomy 21

**DOI:** 10.1186/s12864-017-3993-y

**Published:** 2017-09-12

**Authors:** Ji Hyae Lim, You Jung Han, Hyun Jin Kim, Dong Wook Kwak, So Yeon Park, Sun-Hee Chun, Hyun Mee Ryu

**Affiliations:** 1 Laboratory of Medical Genetics, Medical Research Institute, Cheil General Hospital and Women’s Healthcare Center, Seoul, South Korea; 2 Department of Obstetrics and Gynecology, Cheil General Hospital and Women’s Healthcare Center, Dankook University College of Medicine, Seoul, South Korea; 3 Department of Obstetrics & Gynecology, Division of Maternal-Fetal Medicine, Ewha Womans University, School of Medicine, Seoul, South Korea

**Keywords:** Trisomy 21, Placenta, Gene expression, Microarray

## Abstract

**Background:**

We performed whole human genome expression analysis in placenta tissue (normal and T21) samples in order to investigate gene expression into the pathogenesis of trisomy 21 (T21) placenta. We profiled the whole human genome expression of placental samples from normal and T21 fetuses using the GeneChip Human Genome U133 plus 2.0 array. Based on these data, we predicted the functions of differentially expressed genes using bioinformatics tools.

**Results:**

A total of 110 genes had different expression patterns in the T21 placentas than they did in the normal placentas. Among them, 77 genes were up-regulated in the T21 placenta and 33 genes were down-regulated compared to their respective levels in normal placentas. Over half of the up-regulated genes (59.7%, *n* = 46) were located on HSA21. Up-regulated genes in the T21 placentas were significantly associated with T21 and its complications including mental retardation and neurobehavioral manifestations, whereas down-regulated genes were significantly associated with diseases, such as cystitis, metaplasia, pathologic neovascularization, airway obstruction, and diabetes mellitus. The interactive signaling network showed that 53 genes (40 up-regulated genes and 13 down-regulated genes) were an essential component of the dynamic complex of signaling (*P* < 1.39e-08).

**Conclusions:**

Our findings provide a broad overview of whole human genome expression in the placentas of fetuses with T21 and a possibility that these genes regulate biological pathways that have been involved in T21 and T21 complications. Therefore, these results could contribute to future research efforts concerning gene involvement in the disease’s pathogenesis.

## Background

Trisomy 21 (T21), also known as Down syndrome, results from the total or partial trisomy of chromosome 21 (HSA21). It is the most frequent live-born aneuploidy, affecting 1 in 750 infants [1]. The T21 patients are characterized by a cognitive impairment and may also have muscle hypotonia, dysmorphic features, Alzheimer’s disease (AD), neuropathology, and congenital heart defects [1, 2]. The severity and the phenotypic incidence of T21 vary across patients. Among its possible causes, the genetic (or epigenetic) background of each individual with T21 may contribute to this phenotypic variability.

It is likely that most of the T21 phenotypes are related to alterations in gene expression due to the supernumerary copy of HSA21. According to the ‘gene dosage effect’ hypothesis, some T21 features could be directly explained by the dosage imbalances of genes on HSA21 [3–5]. Therefore, most prior studies have focused on changes in the expression of HSA21 genes in certain tissues (such as fibroblasts [6], whole blood [7], T cells [8], brain [9, 10], and heart [6, 10]) from patients with T21. We also performed a comprehensive survey of genes on HSA21 in placentas of T21 fetuses and profiled expression of 207 genes on HSA21 [11]. Among them, 47 genes showed significantly increased expression in the T21 placenta compared to the normal placenta [11]. The increased genes in the T21 placenta were significantly associated with T21 and T21 complications such as mental retardation, neurobehavioral manifestations, and congenital abnormalities [11]. However, T21 phenotypes may also result from the presence of extra genomic material. Recently, other researchers have demonstrated that the extra HSA21 has deleterious effects that can be seen across the entire genome [12]. Gene expression changes in HSA21 can also affect the gene expression of other chromosomes through the modulation of transcription factors, chromatin remodeling proteins, or related molecules [13–15]. Therefore, understanding the whole genomic determinants that contribute to the various phenotypes of T21 is a major objective in T21 research.

In this study, we investigated whole human genome expression in placentas of euploid and T21 fetuses using microarray technology and identified genes that were aberrantly expressed in placentas from fetuses with T21. We also analyzed the biological function and molecular pathways of the identified genes using various bioinformatics tools.

## Methods

### Study subjects

This study was conducted in accordance with the Declaration of Helsinki. Appropriate institutional review board approval was obtained from the Ethics Committee at Cheil General Hospital (#CGH-IRB-2011-85). All patients provided written informed consent for sample collection and subsequent analysis. Pregnant women with normal or T21 fetuses who were treated at the Department of Obstetrics and Gynecology, Cheil General Hospital Korea were recruited between March 2011 and December 2016. All placenta samples were obtained by chorionic villus sampling (CVS) in the first trimester (11 weeks - 13 weeks of gestation) and stored in liquid nitrogen until analysis.

### Cytogenetic analysis for fetal karyotype

Chromosomal analyses of fetal CVS were carried out using standard protocols as in our previous study [11]. The cells were cultured in the AmnioMAX-C100 culture medium (Invitrogen, Carlsbad, CA, USA). Metaphase chromosomes were stained using the GTG banding method. Twenty metaphases per sample were analyzed. All T21 samples used in this study were complete T21 and all normal samples were completely euploid.

### Gene expression profiling using microarray

#### Array hybridizations

Total RNA was extracted from the placentas of normal (*n* = 12) and T21 (*n* = 6) fetuses using the TRI Reagent (Molecular Research Center, Inc., Cincinnati, OH, USA) according to the manufacturer’s instructions. The RNA was purified using the RNeasy MinElute Cleanup Kit (QIAGEN, Hilden, Germany), as recommended by the manufacturers. Quantity and quality of RNA were measured using a NanoVue™ Plus Spectrophotometer (GE, London, UK) and an Agilent 2100 Bioanalyzer (Agilent Technologies, Santa Clara, CA, USA). An RNA integrity number ≥ 7.0 was considered acceptable for the microarray analysis. Therefore, the placental RNAs of normal (*n* = 5) and T21 (*n* = 3) fetuses were used for whole human genome expression array and their hybridizations carried out separately. Gene expression profiles were determined using the Affymetrix GeneChip Human Genome U133 Plus 2.0 Array (Affymetrix Inc., Santa Clara, CA, USA). The array was used to analyze the expression level of over 47,000 transcripts and variants, including 38,500 well-characterized human genes. Eleven pairs of oligonucleotide probes were used to measure the transcription level of each sequence. GeneChips were washed and stained using the Affymetrix Fluidics Station 450 (Affymetrix Inc.) and scanned using the Affymetrix GeneChip Scanner 3000 7G (Affymetrix Inc.).

#### Gene expression analysis

Expression data were extracted from the scanned images using Expression Console 1.3.1 software (Affymetrix Inc.) and analyzed with robust multichip analysis using Affymetrix default analysis settings and global scaling as the normalization method. The trimmed mean target intensity of each array was arbitrarily set to 100. The normalized, and log-transformed intensity values were analyzed using GeneSpring GX 12.6.1 (Agilent Technologies). Genes with *P* values <0.05 were considered to be significantly differentially expressed between the T21 and normal groups. The *P* values were corrected using the Benjamini and Hochberg false discovery rate (FDR) method to control false positive results from multiple testing [16]. The fold change filters included the requirement that the genes be present in at least 1.5-fold of controls. The genes (*P* < 0.05 and FDR < 0.05 with 1.5-fold expression change) were selected as candidates for further analysis.

### Comparison to T21 fetal brain and T21 adult brain

A publicly available microarray dataset of human T21 fetal brains [10] and T21 adult brains [17] was used to compare the results from T21 fetal placenta. Microarray data for brain of T21 and control subjects were downloaded from the Pevsner Laboratory Web site (http://pevsnerlab.kennedykrieger.org/ds_cel_files.htm) and the Gene Expression Omnibus (GEO, http://www.ncbi.nlm.nih.gov/geo/). The dataset was reanalyzed using the methods described above. We analyzed genes that were up-regulated or down-regulated according to presence or absence of T21 among the fetal placenta, fetal brain, and adult brain.

### Quantitative real-time PCR

The TRIzol reagent was used to extract total RNA from the placentas of normal (*n* = 5) and T21 (*n* = 5) fetuses according to the manufacturer’s instructions. The RNA was purified using the RNeasy MinElute Cleanup Kit (QIAGEN, Hilden, Germany) as recommended by the manufacturers. Quantity and quality measurements were made using a NanoVue™ Plus Spectrophotometer (GE, London, UK) and an Agilent 2100 Bioanalyzer (Agilent Technologies, Santa Clara, CA, USA). The PCR reaction solution for synthesis of complementary DNA (cDNA) contained 10 ng of RNA sample, 0.15 μL 100 mm dNTPs, 1 μL reverse transcriptase (10 U/μL), and 0.19 μL RNase inhibitor (20 U/μL) per 9.16 μL total reaction volume. The thermal profile consisted of 16 °C for 30 min, 42 °C for 30 min, and 95 °C for 5 min. Quantitative real-time PCR was carried out in a 20 μL reaction mixture containing 0.5 μL cDNA (10 ng/μL), 10 μL SYBR Green PCR Master Mix (Applied Biosystems, Foster City, California), 0.8 μL primer set, and 8.7 μL of sterile water. Glyceraldehyde-3-phosphate dehydrogenase (*GAPDH*), a housekeeping gene, was used as an internal control. Amplification was followed by 40 cycles of denaturation at 95 °C for 30 s, annealing at 60 °C for 30 s and extension at 72 °C for 30 s on an ABI PRISM 7500 sequence detection system (Applied Biosystems). All PCR reactions were performed in triplicate, and no template controls were included in any run. The primer specificity was confirmed by melting (dissociation) curve analysis. Comparative quantitation of each target gene was performed based on the cycle threshold (Ct), which was normalized against the Ct of *GAPDH* using the ΔΔCT method. Primer sets and melting temperature for quantitative real-time PCR are described in supplemental data.

### Functional annotation analysis of the candidates

The lists of candidate genes were submitted to a functional annotation tool provided by WebGestalt (http://www.webgestalt.org/webgestalt_2013/). Functional annotation analysis of the candidates was performed according to the presence or absence of inclusion of HSA21 candidate genes. Gene ontology (GO) analysis and disease-associated gene analysis were performed. The GO analysis was performed using a statistic hypergeometric test with a significance level of adj*P* < 0.05, a Benjamini and Hochberg multiple test adjustment, and a minimum of two genes. The candidate genes were annotated by the Kyoto Encyclopedia of Genes and Genomes (KEGG) pathway analysis. Next, we used the Search Tool for the Retrieval of Interacting Genes (STRING v. 10.0) database to predict an interactive network of candidate genes. The target genes were considered seed molecules to obtain direct and indirect protein-protein interactions. This database provides information regarding experimental and predicted interactions from varied sources based on their neighborhood, gene fusion, co-occurrence, co-expression, experiments, and literature mining. We constructed an interactive network of candidate genes with a confidence score of 0.4.

### Statistical analysis

The clinical characteristics of the study population were analyzed using the Mann-Whitney U-test and Wilcoxon Signed Ranks test. In all tests, a *P*-value <0.05 was considered statistically significant. Statistical analyses were performed using the Statistical Package for Social Sciences 12.0 (SPSS Inc., Chicago, IL, USA).

## Results

The clinical characteristics of the study population are provided in Table 1. At the time of tissue sampling, there were no significant difference between the two groups with regard to maternal age, gestational age, body mass index, gravidity, nulliparity, and gender ratio of the fetuses (*P* > 0.05 for all).

**Table 1:** Clinical characteristics of the study population

Characteristics	Trisomy 21 (*n* = 8)	Normal (*n* = 10)	*P* value
Maternal Age (years)	36.8 ± 4.4	36.6 ± 3.4	0.897
Gestational age (weeks)	12.1 ± 0.4	12.3 ± 0.5	0.573
Body mass index (kg/m^2^)	22.3 ± 3.0	22.2 ± 2.3	0.897
Gravidity	2.4 ± 0.7	2.6 ± 0.8	0.460
Nullipara (n)	3	3	1.000
Gender-ratio (female:male)	3:5	3:7	1.000

Data are presented as mean ± standard deviation

We analyzed the expression level of over 47,000 transcripts and variants, including 38,500 well-characterized human genes. We identified differentially expressed genes in T21 placenta samples compared with normal placenta samples. According to criteria of gene expression (FDR < 0.05 with 1.5-fold expression change), 110 genes had significant expression differences between T21 and normal placentas (Supplemental data). Seventy-seven genes were up-regulated in T21 placenta samples, while 33 genes were down-regulated (Table 2). The chromosomal distributions of the candidate genes are shown in Table 2. Over half (59.7%) of the up-regulated genes in T21 were located on HSA21. In contrast, the down-regulated genes were distributed on various chromosomes, not including HSA21-derived genes.

**Table 2:** Candidate genes that are differentially expressed in T21 placentas

Chr.	Up regulation			Down regulation		
	No.	(%)	Gene symbol	No.	(%)	Gene symbol
1	6	7.8	*ATP1A4, C1orf106, INADL, KIF26B, MCOLN3, SYTL1*	3	9.1	*HEYL, NBPF8, WLS*
2	3	3.9	*FZD5, OBSL1, ZNF514*	1	3.0	*PXDN*
3	1	1.3	*ZNF717*	5	15.2	*ADAMTS9, GOLIM4, NCEH1, PAQR9, RICKLE2*
4	2	2.6	*ANKRD37, FRAS1*	3	9.1	*AGA, C4orf32, SLC7A11*
5	0	0.0	*-*	2	6.1	*F2RL1, TICAM2*
6	0	0.0	*-*	2	6.1	*CDKAL1, MAP3K5*
7	3	3.9	*DLX6-AS1, LEP, MET*	1	3.0	*HGF*
9	1	1.3	*STXBP1*	1	3.0	*LAMC3*
10	1	1.3	*CPXM2*	2	6.1	*MMRN2, MRPL43*
11	1	1.3	*MPZL3*	2	6.1	*JAM3, NRGN*
12	2	2.6	*MBD6, OLR1*	2	6.1	*MMP19, PHLDA1*
13	2	2.6	*FLT1, METTL21C*	1	3.0	*EFNB2*
15	1	1.3	*CAPN3*	1	3.0	*ANPEP*
16	2	2.6	*MYLK3, PDXDC1*	1	3.0	*LITAF*
17	2	2.6	*KSR1, WSB1*	1	3.0	*GJC1*
19	3	3.9	*RDH13, ZNF331, ZNF614*	0	0.0	*-*
21	46	59.7	*ADAMTS1, ADAMTS5, AGPAT3, APP, ATP5J, ATP5O, BACE2, BACH1, BRWD1, BTG3, C21orf33, C21orf91, C2CD2, CCT8, CRYZL1, CSTB, DONSON, DYRK1A, ETS2, GABPA, GART, GCFC1, HSPA13, HUNK, IGSF5, LTN1, MCM3AP, MIS18A, MORC3, MRPL39, NDUFV3, NRIP1, PCP4, PSMG1, PTTG1IP, SCAF4, SETD4, SLC37A1, SON, SYNJ1, TRAPPC10, TTC3, U2AF1, UBE2G2, USP16, USP25*	0	0.0	*-*
22	1	1.3	*H1F0*	2	6.1	*KDELR3, SUSD2*
X	0	0.0	-	3	9.1	*COL4A5, GPC4, GPRASP2*
Total	77	100.0		33	100.0	

*Chr* chromosome, *No* number, *T21* trisomy 21

**Fig. 1:**
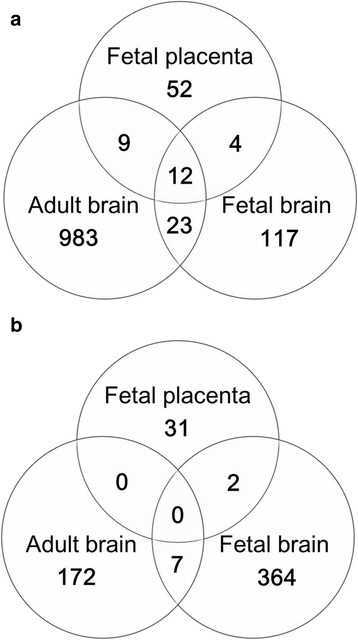
Comparison of differentially expressed genes in adult brain, fetal brain, and fetal placenta of T21. The up-regulated genes in the adult brains, fetal brains, and fetal placentas with T21 were 1027, 156, and 77, respectively (**a**). Of the up-regulated genes, 12 (*APP, C21orf33, C2CD2, CSTB, HSPA13, LTN1, MORC3, MRPL39, NRIP1, PTTG1IP, TRAPPC10, USP16*) were commonly up-regulated in all tissues, regardless of tissue types and developmental stages (**a**). The down-regulated genes in the adult brains, fetal brains, and fetal placentas with T21 were 179, 373, and 33, respectively (**b**). None of the down-regulated genes were commonly down-regulated across the tissue types and developmental stages. Two genes (*CDKAL1* and *GJC1*) were down-regulated in both fetal brains and fetal placentas (**b**)

Additionally, we compared the expression data from adult and fetal brain tissue to further investigate the gene expression changes observed in T21. A publicly available dataset of fetal and adult human brains was reanalyzed according to the criteria of this study (FDR < 0.05 with 1.5-fold expression change). In adult brains with T21, 1027 genes were up-regulated and 179 genes were down-regulated compared to their respective expression patterns in normal adult brains (Fig. 1a). In fetal brains with T21, 156 genes were up-regulated and 373 genes were down-regulated (Fig. 1b). Of the up-regulated genes in the adult brains, fetal brains, and fetal placentas with T21, 12 (*APP, C21orf33, C2CD2, CSTB, HSPA13, LTN1, MORC3, MRPL39, NRIP1, PTTG1IP, TRAPPC10, USP16*) were commonly up-regulated regardless of tissue type or developmental stage (Fig. 1a). These 12 genes were all located on HSA21. In contrast, none of the down-regulated genes were commonly down-regulated across the tissue types and developmental stages. Two genes (*CDKAL1* and *GJC1*) were commonly down-regulated in both fetal brains and fetal placentas (Fig. 1b).

To confirm expression changes of array, we performed quantitative real-time PCR. We randomly selected 10 up-regulated genes, 6 down-regulated genes, 3 non-significant genes based on microarray data. The *GAPDH* was used as a reference gene. We compared their mRNA levels between T21 and euploid samples. The expression patterns of genes were consistent with array data (Table 3).

**Table 3:** Quantitative real-time PCR results for selected genes

Gene symbol	Chromosome number	Expression change (fold)		*P* value
		Microarray	qPCR	
Up-regulated genes in trisomy 21				
*ANKRD37*	4	2.064	4.312	0.036
*FLT1*	13	2.272	5.599	0.002
*HUNK*	21	2.041	2.189	0.013
*IGSF5*	21	2.310	2.014	0.017
*LEP*	7	5.840	2.867	0.024
*MCOLN3*	1	2.220	7.137	0.006
*METTL21C*	13	2.905	4.585	0.010
*MPZL3*	11	2.243	8.906	0.023
*SON*	21	1.970	4.480	0.032
*SYNJ1*	21	1.984	5.653	0.025
Down-regulated genes in trisomy 21				
*COL4A5*	X	2.465	5.672	0.003
*GJC1*	17	2.188	3.714	0.019
*GOLIM4*	3	2.335	2.857	0.005
*HGF*	7	2.242	2.787	0.013
*MRPL43*	10	2.611	2.188	0.015
*SUSD2*	22	2.324	4.908	0.001
Non-significant genes				
*PLEK*	2	0.874	0.757	0.273
*MAPKAP1*	9	0.970	0.784	0.499
*ATP6*	11	1.075	0.838	0.613

Data were normalized to the *GAPDH* housekeeping gene. Values for microarray data are fold intensity. For each quantitative PCR experiment, values were determined by measuring samples in triplicate (mean ± standard deviation). Each experiment was performed independently at least three times. *P* values are derived from quantitative real-time PCR data

We performed functional annotation analysis of the genes that were differentially expressed in T21 according to the presence or absence of inclusion of HSA21 candidate genes. In GO analysis of the 110 candidate genes in whole genome, the genes were analyzed in all categories including biological process (BP), cellular component (CC), and molecular function (MF) (Table 4). In the BP category, the most statistically significant associations were found in hydrogen peroxide-mediated programmed cell death (raw*P* = 2.65e-07, adj*P* = 2.01e-04). The arginine/serine domain binding and peptidase activity (raw*P* = 8.00e-04, adj*P* = 4.21e-02) in the MF category, as well as the extracellular matrix (raw*P* = 3.64e-05, adj*P* = 4.70e-04) in the CC category were most significantly associated with the candidate genes. In addition, GO analysis was performed with the 64 candidates of chromosomes other than HSA21. In the BP category, the most statistically significant associations were found in hydrogen peroxide-mediated programmed cell death such as results in whole genome (raw*P* = 4.75e-08, adj*P* = 3.28e-05). However, there were no genes in the MF category with statistically significant changes in transcript level. In the CC category, the proteinaceous extracellular matrix category was the most significantly associated with the candidate genes (raw*P* = 2.00e-04, adj*P* = 5.90e-03).

**Table 4:** GO analysis of candidate genes

	Pathway	Gene symbol		rawP	adjP
		Up regulation in T21	Down regulation in T21		
Whole genome					
BP	Hydrogen peroxide-mediated programmed cell death	*MET*	*HGF, MAP3K5*	2.65e-07	2.01e-04
	Ovulation from ovarian follicle	*ADAMTS1, LEP, NRIP1*	*MMP19*	7.94e-07	4.00e-04
	Negative regulation of hydrogen peroxide-mediated programmed cell death	*MET*	*HGF*	4.17e-05	7.60e-03
	Striated muscle cell differentiation	*APP, CAPN3, MET, MYLK3,OBSL1*	*COL4A5, EFNB2*	4.00e-04	4.54e-02
MF	Arginine/serine domain binding	*SON, U2AF1*	*-*	8.00e-04	4.21e-02
	Peptidase activity	*ADAMTS1, ADAMTS5, BACE2, CAPN3, CPXM2, USP16, USP25*	*ADAMTS9, ANPEP, HGF, MMP19*	8.00e-04	4.21e-02
	Metallopeptidase activity	*ADAMTS1, ADAMTS5, CPXM2*	*ADAMTS9, ANPEP, MMP19*	1.00e-03	4.21e-02
	Glucocorticoid receptor binding	*ETS2, NRIP1*	*-*	1.40e-03	4.28e-02
CC	Extracellular matrix	*ADAMTS1,ADAMTS5, CPXM2, FRAS1,*	*ADAMTS9, COL4A5, GPC4, LAMC3, MMP19, MMRN2, PXDN*	3.64e-05	4.70e-04
	Mitochondrial proton-transporting ATP synthase complex, coupling factor	*ATP5J, ATP5O*	*-*	1.20e-03	4.61e-02
	Extracellular region part	*ADAMTS1, ADAMTS5, CPXM2, FLT1, LEP, MET*	*ADAMTS9, PXDN, COL4A5, GPC4, HGF, JAM3, LAMC3, MMP19, MMRN2*	1.60e-03	4.61e-02
	Basement membrane	*ADAMTS1*	*COL4A5, LAMC3, MMRN2*	1.80e-03	4.61e-02
Chromosomes other than HSA21					
BP	Hydrogen peroxide-mediated programmed cell death	*MET*	*HGF, MAP3K5*	4.75e-08	3.28e-05
	Anatomical structure formation involved in morphogenesis	*CAPN3, FLT1, FZD5, KIF26B, MET, MYLK3, OBSL1, STXBP1*	*ANPEP, COL4A5, EFNB2, GJC1, HEYL, HGF, JAM3, MMP19, MMRN2, WLS,*	8.78e-06	2.30e-03
	Regulation of intracellular protein kinase cascade	*CAPN3,FLT1, FZD5, LEP, MET*	*F2RL1, HGF, LITAF, MAP3K5, TICAM2, WLS,*	3.23e-05	3.70e-03
	Cell differentiation	*CAPN3,FLT1, FZD5, MCOLN3, MET, MYLK3, LEP, OBSL1, STXBP1*	*ADAMTS9, ANPEP, COL4A5, EFNB2, F2RL1, GJC1, HEYL, HGF, JAM3, LAMC3, MMP19, PRICKLE2, SLC7A11*	9.31e-05	4.90e-03
	Circulatory system development	*FLT1, FZD5, GJC1, JAM3, MYLK3, OBSL1*	*ANPEP, EFNB2, HEYL, MMP19, MMRN2*	7.93e-05	4.90e-03
CC	Proteinaceous extracellular matrix	*-*	*ADAMTS9, COL4A5, GPC4, LAMC, MMP19, MMRN2, PXDN*	2.00e-04	5.90e-03
	Extracellular space	*CPXM2, FLT1, JAM3, LEP, MET,*	*ADAMTS9, GPC4, HGF, MMRN2, PXDN*	5.00e-04	1.11e-02

*HSA21* huma﻿n chromosom﻿e 21, *BP* biological process, *MF* molecular function, *CC* cellular component, *T21* trisomy 21

rawP: *p* value from hypergeometric test, adjP: *p* value adjusted by the multiple test adjustment

In KEGG analysis of the 110 candidate genes in whole genome, various pathways such as those of focal adhesion, AD, and Parkinson’s disease were significantly associated with the differently expressed genes in T21 (both raw*P* and adj*P* < 0.001 in all, Table 5). In KEGG analysis of the 64 candidate genes in chromosomes other than HSA21, focal adhesion, cancer pathway, and cytokine-cytokine receptor interaction were found statistically significant pathway (both raw*P* and adj*P* < 0.01 in all, Table 5). These pathways were found in KEGG analysis of the candidates in whole genome including HSA21.

**Table 5:** Pathway analysis of candidate genes

Pathway name of KEGG	Gene symbol		rawP	adjP
	Up regulation in T21	Down regulation in T21		
Whole genome				
Focal adhesion	*FLT1, MET, MYLK3*	*COL4A5, HGF, LAMC3*	1.32e-05	3.01e-05
Alzheimer’s disease	*APP,ATP5J,ATP5O, BACE2, NDUFV3*	*-*	7.23e-05	9.15e-05
Parkinson’s disease	*ATP5J, ATP5O, NDUFV3, UBE2G2*	*-*	4.00e-04	3.24e-03
Pathways in cancer	*FZD5, MET*	*COL4A5, HGF, LAMC3*	1.51e-03	7.21e-03
Cytokine-cytokine receptor interaction	*FLT1, LEP, MET*	*HGF*	4.79e-03	1.44e-02
Metabolic pathways	*AGPAT3, ATP5J, ATP5O, GART, NDUFV3, SYNJ1*	*ANPEP*	2.59e-02	3.45e-02
Chromosomes other than HSA21				
Focal adhesion	*FLT1, MET, MYLK3*	*COL4A5, HGF, LAMC3*	5.53e-07	9.95e-06
Pathways in cancer	*FZD5, MET*	*COL4A5, HGF, LAMC3*	1.00e-04	9.00e-04
Cytokine-cytokine receptor interaction	*FLT1, LEP, MET*	*HGF*	7.00e-04	4.20e-03

*HSA2﻿1* human ch﻿romosome 21, *T21* trisomy 21, rawP: *p* value from hypergeometric test, adjP: *p* value adjusted by the multiple test adjustment

The disease association of the candidate genes in whole genome was analyzed separately according to expression pattern in T21 (Table 6). In the up-regulated candidate genes, the most statistically significant association was identified in T21 (raw*P* = 6.04e-34, adj*P* = 5.80e-32). Many genes were significantly associated with T21 complications such as chromosome disorders, mental retardation, and neurobehavioral manifestations. In the down-regulated candidate genes, *GJC1* and *TICAM2* demonstrated the most statistically significant association with cystitis (raw*P* = 6.77e-05, adj*P* = 3.40e-04). *ANPEP, EFNB2* and *HGF* were significantly associated with metaplasia and pathologic neovascularization (raw*P* and adj*P* < 0.01 in all). Other genes were significantly associated with airway obstruction and diabetes mellitus (raw*P* and adj*P* < 0.05 in all). In disease analysis of the 64 candidate genes in chromosomes other than HSA21, pathologic neovascularization showed the most statistically significant association with the differently expressed genes in T21 (raw*P* = 2.88e-06, adj*P* = 3.00e-04). Unlike the results of disease association analysis in whole genome, gestational diabetes and vascular disease were significantly associated (raw*P* and adj*P* < 0.01 in all, Table 6).

**Table 6:** Disease association of candidate genes

Disease	Gene symbol	rawP	adjP
Whole genome			
Up-regulated genes in trisomy 21			
Trisomy21	*APP, ATP5O, BACE2, BACH1, BRWD1, BTG3, C2CD2, C21orf33, CRYZL1, DONSON, DYRK1A, ETS2, GART, GCFC1, NDUFV3, MCM3AP, MIS18A, PCP4, PSMG1, SETD4, SON, TTC3, UBE2G2,USP25*	6.04e-34	5.80e-32
Chromosome disorders	*APP, BACE2, BACH1, BRWD1, C2CD2, C21orf33, CRYZL1, DYRK1A, ETS2, GART, PCP4, PSMG1, SETD4, SYNJ1, TRAPPC10, TTC3, UBE2G2, USP25*	3.83e-20	1.84e-18
Mental retardation	*APP, BACE2, BACH1, BRWD1, C2CD2, CRYZL1, DYRK1A, ETS2, GART, PCP4, PSMG1, SETD4, STXBP1, TTC3, UBE2G2, USP25*	4.89e-17	1.56e-15
Neurobehavioral manifestations	*APP, BACE2, BACH1, BRWD1, C2CD2, CRYZL1, DYRK1A, ETS2, GART, PCP4, PSMG1, SETD4, SON, TTC3,UBE2G2, USP25*	2.63e-15	6.31e-14
Trisomy	*APP, BACH1, C2CD2, DYRK1A, ETS2, PCP4, PSMG1, TTC3,*	2.17e-11	4.17e-10
Down-regulated genes in trisomy 21			
Cystitis	*GJC1, TICAM2*	6.77e-05	3.40e-04
Metaplasia	*ANPEP, EFNB2, HGF*	3.00e-04	5.01e-03
Pathologic neovascularization	*ANPEP, EFNB2, HGF*	2.00e-04	5.00e-03
Airway obstruction	*GPC4, WLS*	6.00e-04	7.50e-03
Diabetes mellitus	*ADAMTS9, C4orf32, CDKAL1*	1.00e-03	1.00e-02
Chromosomes other than HSA21			
Pathologic Neovascularization	*ANPEP, FLT1, EFNB2, HGF, MET*	2.88e-06	3.00e-04
Gestational diabetes	*ADAMTS9, CDKAL1, FLT1, LEP,*	3.76e-06	3.00e-04
Metaplasia	*ANPEP, EFNB2, FLT1, HGF, MET*	5.67e-06	3.00e-04
Cystitis	*GJC1, TICAM2*	3.00e-04	5.40e-03
Vascular Diseases	*FLT1, HGF, LEP, NCEH1, OLR1*	3.00e-04	5.40e-03

*HSA21* human chromosome 21, rawP: *p* value from hypergeometric test, adjP: *p* value adjusted by the multiple test adjustment

**Fig. 2:**
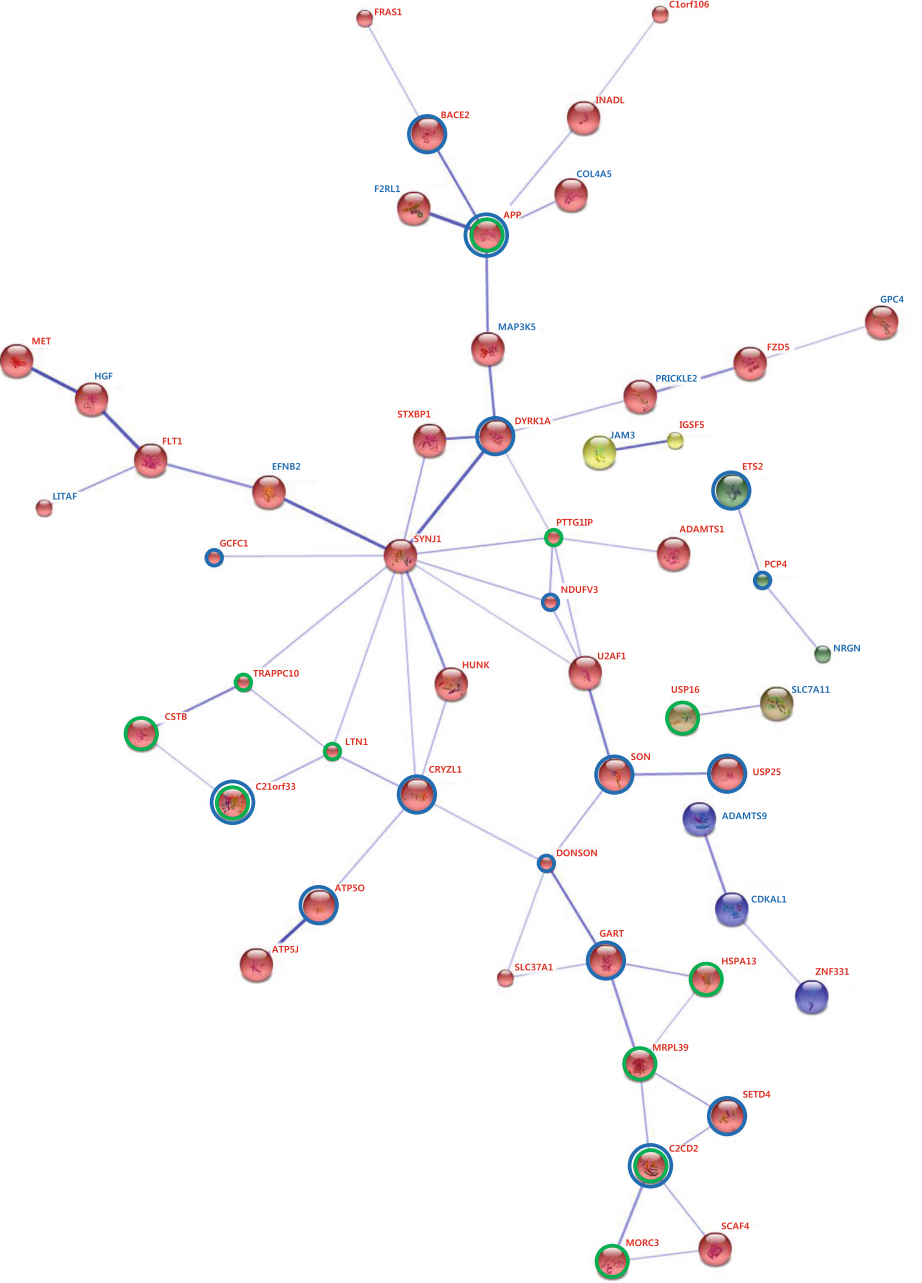
Interaction networks of genes that are differentially expressed in T21 placentas and euploid placentas. The list of the identified genes was subjected to STRING (v. 10.0) analysis to reveal functional interactions. Each *node* represents a protein, and each *edge* represents an interaction. *Thicker lines* represent stronger associations. *Red letters* and *blue letters* present up-regulated genes and down-regulated genes, respectively, in the fetal placentas with T21. *Green circles* represent genes that are concurrently up-regulated in the adult brain, fetal brain, and fetal placenta with T21. *Blue circles* represent genes that were significantly associated with T21 (*P* < 1.46e-34)

The interactive signaling networks were constructed using genes that were differentially expressed in the T21 placenta compared to the normal placenta. Based on 77 genes that were up-regulated in T21 placentas, the interaction network has significance beyond statistical data. Thirty-four genes made up the dynamic complex of signaling (*P* < 3.88e-09, data not shown). With regard to the 33 down-regulated genes in T21 placentas, there was only one expected edges between ADAMTS9 and CDKAL1 (*P* < 0.75, data not shown). The interaction network has significance beyond the statistical data with regard to all 110 genes that were differentially expressed between T21 and normal placentas. Fifty-three genes (40 up-regulated genes and 13 down-regulated genes) were an integral part of the dynamic complex of signaling under a confidence score of 0.4 (*P* < 1.39e-08, Fig. 2). In the interactive signaling networks, various down-regulated genes act as connecting nodes in the dynamic complex of up-regulated genes. Of the 53 interacting genes, 11 were commonly up-regulated in the adult brain, fetal brain, and fetal placenta with T21 (green circle). Sixteen interacting genes were significantly associated with T21 (blue circle, *P* < 1.46e-34). One major cluster of the interactive signaling networks was identified and consisted of 35 up-regulated and 8 down-regulated genes. Center gene of the cluster was synaptojanin 1(*SYNJ1*) that was connected with 11 genes including *STXBP1, DYRK1A, PTTG1IP, NDUFV3, U2AF1, HUNK, CRYZL1, LTN1, TRAPPC10, GCFC1,* and *EFNB2*.

## Discussion

T21 is caused by an extra copy of all or part of HSA21. The main etiology of this disease is thought to result from the potential implications of imbalanced expression of genes on HSA21 [3–5]. Most studies have confirmed a primary gene dosage effect of HSA21 in T21. However, the downstream consequences of T21 are complex. Therefore, in addition to primary gene dosage effects, secondary (downstream) effects on disomic genes are also likely to play a major role in T21. On HSA21, gene expression may be regulated by dosage compensation or other mechanisms. Therefore, only a subset of those genes is expressed at the expected 50% increased levels. For genes assigned to chromosomes other than HSA21, the effect of T21 could either be relatively subtle or massively disruptive. Gene expression changes in HSA21 are likely to affect the gene expression on other chromosomes through the modulation of transcription factors, chromatin remodeling proteins, or related molecules [15–17]. Another study about transcriptome analysis of monozygotic twins discordant for T21 identified the existence of chromosomal domains of gene expression dysregulation between trisomic and normal fibroblasts [12]. These results suggested that the nuclear compartments of trisomic cells undergo modifications of the chromatin environment affecting the overall transcriptome and that gene expression dysregulation domains may have an influence on some T21 phenotypes [12]. Therefore, recent studies have suggested that the trisomy has effects on the expression of disomic genes. Understanding the whole genomic determinants that contribute to the various phenotypes of T21 has become a major objective in T21 research.

In this study, we investigated the whole genome of placentas from human fetuses with T21 compared to those of euploid fetuses. We found 110 genes that were differently expressed in the T21 placenta compared to in the euploid fetuses. Among them, 77 genes were up-regulated and 33 were down-regulated in the T21 placenta compared to their respective expression levels in euploid placentas. More than half of the up-regulated genes (59.7%, *n* = 46) are located on HSA21, whereas the down-regulated genes are located on various chromosomes, not including HSA21. Especially, 12 genes on HSA21 (including *APP, C21orf33, C2CD2, CSTB, HSPA13, LTN1, MORC3, MRPL39, NRIP1, PTTG1IP, TRAPPC10, USP16*) were commonly up-regulated in the adult brains, fetal brains, and fetal placentas with T21, regardless of tissue type or developmental stage. We also found that various genes on chromosomes other than HSA21 were up-regulated or down-regulated in the T21 placentas. Additionally, the results of functional annotation analysis using candidates of whole genome were included all those according to the presence or absence of HSA21 candidate genes. These results suggest that the whole genomic imbalance in T21 may have an influence on the various phenotypes of T21.

In addition, we identified several gene clusters associated with T21 using in-silico pathway-based exploratory analysis of genes with expressions specific to T21 placentas. These clusters demonstrated an association between up-regulated genes and down-regulated genes in T21 placentas. In our study, network of the 33 down-regulated genes was only found between *ADAMTS9* and *CDKAL1*. However, the 13 down-regulated genes were part of the network of 110 genes specific to T21 placentas. These genes acted as a connecting node of disconnected clusters in the network of up-regulated genes. Therefore, we predicted one major cluster that consisted of 35 up-regulated genes and 8 down-regulated genes (red node, Fig. 1). The center gene of the cluster was synaptojanin 1(*SYNJ1*), which was connected with the following 11 genes: *CRYZL1, DYRK1A, EFNB2, GCFC1, HUNK, LTN1, NDUFV3, PTTG1IP, STXBP1, TRAPPC10,* and *U2AF1*. The *SYNJ1* is located on HSA21 and is coding the synapse associated protein that is of key interest in T21. The gene is present in triplicate in T21 [18]. SYNJ1 is a brain enriched phosphoinositide phosphatase [19] that is involved with endocytosis and synaptic vesicle cycling [20, 21]. A proper dosage of this gene is required for proper synaptic activity. Several studies using immunocytochemical or western blotting approaches have identified increased SYNJ1 protein levels in the DS brain [22], particularly within the frontal cortex [23]. Interestingly, our network shows the possibility of various new processes, including SYNJ1, in the molecular mechanisms related to the pathophysiology of T21. First, SYNJ1 could be related to APP via DYRK1A and MAP3K5. T21 patients, who carry a triplication of both SYNJ1 and APP, develop early-onset AD [24]. This could be the result of overexpressed APP alone; however, some lines of evidence argue in favor of the combined effects of these two genes in AD development. The beneficial impact of SYNJ1 reduction in AD was confirmed in a mouse model of AD [25]. In these animals, the hemizygous deletion of SYNJ1 rescued deficits in learning and memory. This protective effect is a result of a decrease in amyloid plaque burden mediated through accelerated endosomal/lysosomal degradation of A*β* [26]. These data underline the potential of SYNJ1 reduction as a possible therapeutic strategy to counteract AD pathology. Our network shows that SYNJ1 and APP in T21 could be simultaneously regulated by the up-regulation of DYRK1A and down-regulation of MAP3K5. This connection might provide new insight into the pathophysiology related with SYNJ1 and APP in T21 with AD. In addition, SYNJ1 could be associated with genes that play roles in mitochondrial function such as two genes encoding subunits of ATP synthase (*ATP5O* and *ATP5J*) and mitochondrial ribosomal protein L39 (*MRPL39*) via *CRYZL1*. The expression levels of these genes were increased in our microarray experiments, as well as in a previous study [10]. Additionally, various mitochondrion-related functional groups were significantly regulated. The abnormal regulation of these transcripts and functional groups could explain the impaired mitochondrial function that has been observed in T21 [27]. Overall, our findings warrant further studies addressing these new clusters of genes associated with the pathogenesis of T21. However, a lot of our results were based on databases of bioinformatics tools. Although these in-silico bioinformatics’ tools are useful to predict the new insight of multi genes associated with the pathophysiology of disorder, these in-silico results could be not strong data to justify the functional significance of genes. Moreover, this study was limited by its small sample size. Therefore, a larger scale study is needed to provide enough evidence to highlight the functional significance of the identified genes in the pathophysiology of T21.

## Conclusions

To our knowledge, this is the first study to comprehensively survey the whole human genomes from placentas of T21 fetuses. This study identified 110 genes that were differentially expressed in euploid fetuses and those with T21. Our results demonstrate that these genes may regulate the many biological pathways that have been implicated in T21 and its complications are possibly regulated by these genes. Therefore, this work provides a variety of information that contributes to a better understanding of the molecular mechanisms and biological pathways of T21.

## Abbreviations

AD: Alzheimer’s disease
BP: Biological process
CC: Cellular component
CVS: Chorionic villus samples
FDR: False discovery rate
GO: Gene ontology
HSA21: Human chromosome 21
KEGG: Kyoto encyclopedia of genes and genomes
MF: Molecular function
T21: Trisomy 21
